# A rare case of dyskeratosis congenita with DKC1 mutation presenting initially as thrombocytopenia: Case report

**DOI:** 10.1097/MD.0000000000046956

**Published:** 2026-01-02

**Authors:** Ruifan Wen, Jidong Tian

**Affiliations:** a Medical School, Hunan University of Chinese Medicine, Changsha, China; b Department of Gastroenterology, The Second Xiangya Hospital of Central South University, Changsha, China.

**Keywords:** bone marrow failure, case report, DKC1, dyskeratosis congenita (DC), thrombocytopenia

## Abstract

**Rationale:**

Dyskeratosis congenita is a rare genetic disorder classically characterized by the mucocutaneous triad and bone marrow failure. Diagnosis is challenging when hematologic manifestations precede classic features.

**Patient concerns:**

A 10-year-old male initially presented with isolated thrombocytopenia, which was misdiagnosed as aplastic anemia.

**Diagnoses:**

Retrospective examination revealed the mucocutaneous triad. Genetic testing confirmed a pathogenic hemizygous DKC1 mutation (c.1058C>T, p.A353V).

**Interventions:**

The patient received a 3-month course of androgen therapy. Genetic counseling and prenatal testing were conducted for the family.

**Outcomes:**

Androgen therapy maintained platelets at 30–60 × 10^9^/L without bleeding or adverse events during follow-up. The asymptomatic mother was a heterozygous carrier. Prenatal testing identified the same mutation in a male fetus, leading to pregnancy termination at 24 weeks.

**Lessons:**

Dyskeratosis congenita can present with isolated cytopenia, risking misdiagnosis. Early genetic confirmation is vital for management and enables informed reproductive planning through prenatal diagnosis. Androgen therapy may be an effective supportive treatment.

## 1. Introduction

Dyskeratosis congenita (DC), also known as Zinsser–Engman–Cole syndrome, is a rare inherited disorder with a prevalence of 1 in 1 million individuals.^[1]^ It is a congenital syndrome involving both mesodermal and ectodermal dysplasia and exhibits considerable clinical and genetic heterogeneity. The condition is characterized by a classic mucocutaneous triad: abnormal skin pigmentation, nail dystrophy, and oral leukoplakia. In addition to these hallmark features, DC is often complicated by progressive bone marrow failure (BMF), pulmonary fibrosis, and an elevated risk of malignancies – particularly squamous cell carcinomas (SCCs) and hematological cancers.^[2]^ Diagnosis typically relies on characteristic clinical findings and laboratory tests, with confirmation through genetic analysis. Variability in age of onset and severity of mucocutaneous and systemic involvement can lead to misdiagnosis as other BMF syndromes, such as aplastic anemia or Fanconi anemia. In some severe cases, patients die prematurely before mucocutaneous signs become apparent, further complicating timely detection. Here, we report the case of a 10-year-old boy with X-linked DC who was initially managed as a case of aplastic anemia.

## 2. Case report

A 10-year-old male patient was admitted on March 19, 2015, reporting intermittent dizziness and fatigue of more than 1 year’s duration. The symptoms had begun insidiously and were occasionally accompanied by gingival bleeding and epistaxis. The patient had not sought medical care at symptom onset. A complete blood count from July 2014 had previously revealed thrombocytopenia. A subsequent bone marrow trephine biopsy showed marked megakaryocyte hypoplasia – with complete absence in examined sections – along with foci of non-hematopoietic cell clusters, findings consistent with early-stage aplastic anemia (AA). A provisional diagnosis of atypical AA was established. The patient was started on cyclosporine and compound medications, with regular blood count monitoring. Platelet counts remained persistently low, ranging from 30 × 10^9^/L to 60 × 10^9^/L, although neutrophil and hemoglobin levels were within normal limits. His medical history included recurrent infections, such as upper respiratory tract infections and diarrhea, as well as occasional dysuria and epiphora. He was the first-born child with a birth weight of 2.4 kg and had otherwise experienced normal growth and development. There was no significant family history of known hereditary disorders.

**Figure 1.**
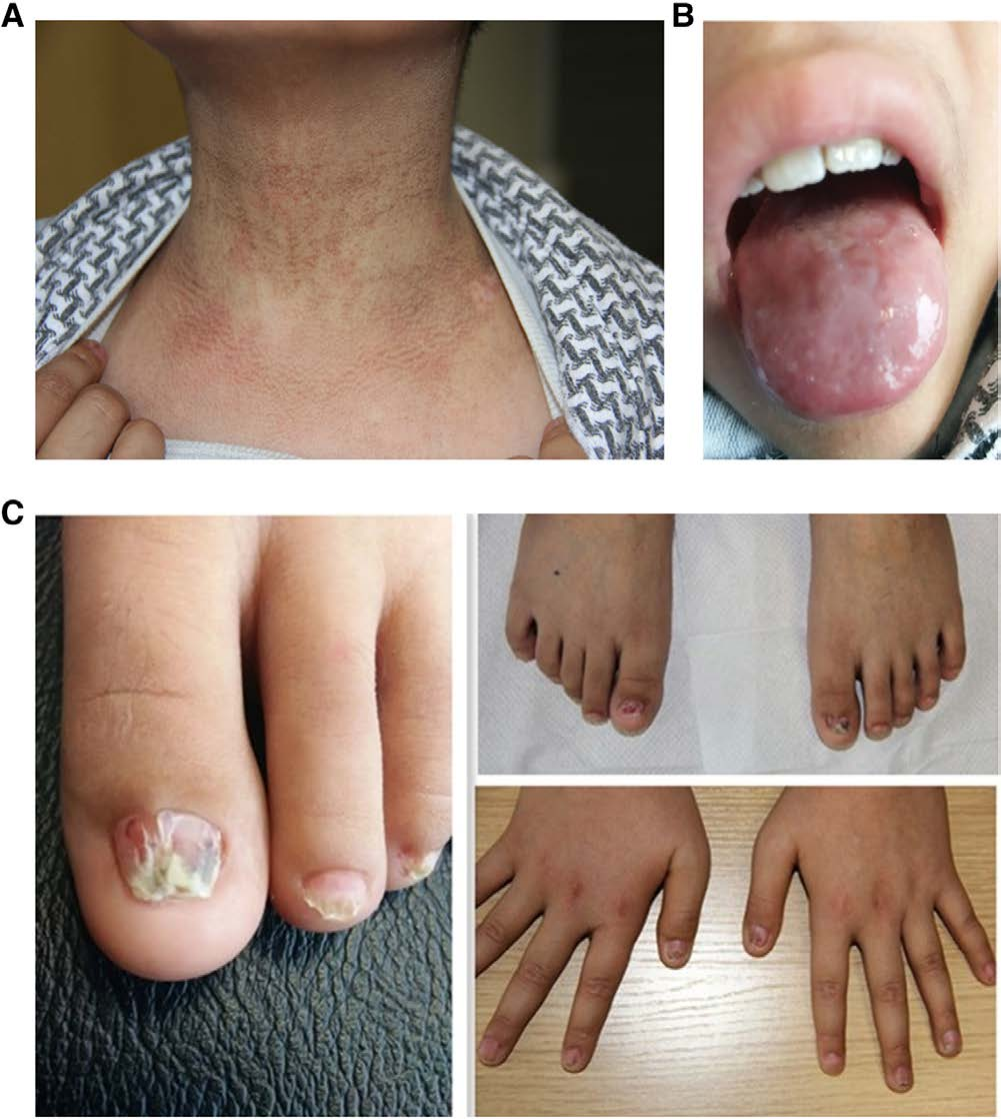
The clinical features of the index case showing grayish brown pigmentation over his neck (A), leukoplakia on the blade of his tongue (B), and dysplasia, atrophy or thinning, partial gryphosis or ptosis of his nails and toe-nails (C).

Physical examination revealed reticulated brown-gray pigmentation was observed on the neck (Fig. 1A), along with hirsutism on the extremities. Mucosal leukoplakia was noted on the dorsal surface of the tongue (Fig. 1B). Nail changes included dystrophy, atrophy, thinning, partial curling, and loss (Fig. 1C). Hyperkeratosis was observed on the plantar surfaces. Further history-taking disclosed that the patient had developed atrophic changes and partial loss of fingernails and toenails as early as 2013, followed by the gradual appearance of skin hyperpigmentation on the anterior neck in 2014. However, these cutaneous and ungual abnormalities had not been addressed during prior clinical evaluations. Laboratory studies at admission were as follows: WBC 3.03 × 10^9^/L, hemoglobin 115 g/L, RBC 3.53 × 10^12^/L, and platelets 35 × 10^9^/L. Following written informed consent, bone marrow aspiration and trephine biopsy were performed. Histopathological evaluation of an adequate trephine specimen demonstrated: hypoproliferative bone marrow with overall reduced cellularity; intact granulopoiesis showing full maturation; focal erythroid hyperplasia, mainly in mid- to late-stage forms; readily identifiable lymphocytes; complete absence of megakaryocytes in all sections; and no overtly abnormal or malignant cells. Bone marrow silver staining was negative.

## 3. Diagnostic assessment

**Figure 2.**
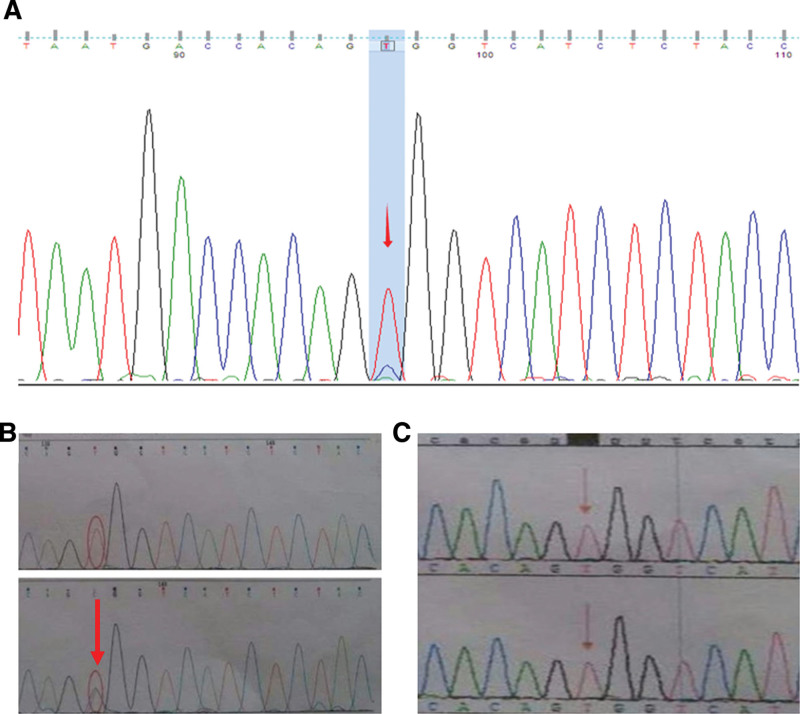
Sanger sequencing chromatograms of the DKC1 gene mutation (c.1058C > T) on exon 11 of the DCK1 gene. (A) Proband showing hemizygous mutation (arrow indicates the T peak at position c.1058); (B) Comparison of the proband (upper) and mother (lower): the mother is heterozygous with both C and T peaks at c.1058 (arrow); (C) Comparison of the proband (upper) and male fetus (lower): the fetus carries the same hemizygous mutation (arrow).

**Figure 3.**
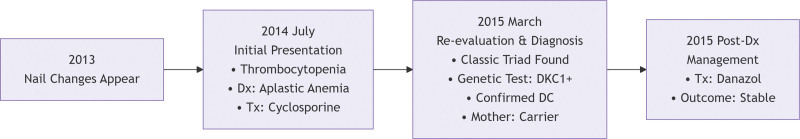
Diagnostic timeline of the proband with DC. DC = dyskeratosis congenital.

Following a thorough clinical and family history assessment, genetic testing was initiated. With parental consent, peripheral blood samples were obtained for analysis of genes associated with DC. Using PCR and sequencing, the coding regions and flanking transcriptional sites of eight genes (*DKC1*, *TERC*, TERT, *NOP10*, *NHP2*, *TINF2*, *C16orf57*, and *TCAB1*) were examined. Sequencing revealed a missense mutation in exon 11 of DKC1, specifically c.1058C > T (p.Ala353Val) (Fig. 2A). Familial segregation analysis confirmed the mother as a heterozygous carrier of the same variant (Fig. 2B), while the father did not. Telomere length testing was not performed due to limited conditions. Based on the patient’s typical clinical presentation and the results of genetic testing, and excluding other causes of BMF such as infections and tumors, the patient was diagnosed with DC. The diagnostic workflow is summarized in Figure 3.

The patient then received androgen therapy and supportive care. The choice of danazol was based on several considerations: the patient’s relatively stable clinical condition despite thrombocytopenia, his young age, the significant toxicity and risks associated with hematopoietic stem cell transplantation (HSCT) in patients with DC, and the substantial financial burden that HSCT would impose on the family. After 3 months of danazol therapy, his platelet counts stabilized at 30 to 60 × 10^9^/L without new bleeding episodes, though transfusion independence was not achieved. This patient had no adverse effects during the follow-up period. Genetic counseling was provided to the family. During the mother’s subsequent pregnancy, amniocentesis was performed for prenatal diagnosis, which confirmed the presence of the same genetic mutation in the male embryo (Fig. 2C). Following this result, the pregnancy was terminated.

## 4. Discussion

DC, also known as Zinsser–Engman–Cole syndrome, is a rare genetic disorder with an incidence of approximately 1 in 1,000,000, with a male-to-female ratio of 13:1.^[3]^ It is a congenital disorder characterized by ectodermal and mesodermal developmental defects. First reported by Zinsser in 1906, the disorder was further detailed by Engman and Cole in 1926 and 1930.^[4]^ DC can be inherited in X-linked recessive, autosomal dominant, or autosomal recessive patterns. To date, at least 19 genes associated with DC have been identified, including *DKC1*, *TERC*, *TERT*, and others, with mutations affecting telomerase activity and leading to telomere dysfunction. Recent genetic studies have identified new loci on known genes and discovered the novel X-linked *POLA1* gene.^[5]^ Despite these advances, approximately 30% of patients with DC still lack a identified pathogenic mutation.

The *DKC1* gene, initially described by Connor et al in 1986 and named by Heiss et al in 1998, encodes dyskerin – a core component of the telomerase complex essential for telomere maintenance and ribosomal RNA pseudouridylation.^[6,7]^ Registry data indicated that *DKC1* is the most frequently mutated gene in DC. Among its mutations, A353V is the most common recurrent variant, accounting for 30 to 40% of all X-linked DC cases.^[8,9]^ Although phenotypic expression of the A353V mutation is variable, it often confers a severe disease course, ranging from classical DC to the more aggressive Hoyeraal–Hreidarsson syndrome (HHS).^[10]^ A literature review identified reports describing the clinical manifestations of patients with DC caused by the A353V mutation.^[10–20]^ The analysis of the 18 documented A353V cases (Table 1) reveals distinct clinical patterns. Among the cases with reported data, the classic mucocutaneous triad was universally present (17/17, 100%), underscoring its paramount diagnostic importance. BMF was a frequent complication, occurring in 66.7% (12/18) of the cohort. Notably, 22.2% (4/18) presented with the severe HHS variant. When comparing our patient to this cohort, his presentation aligns with the most consistent finding – the universal presence of the triad among reported cases – and the common complication of BMF, but lacks the severe HHS features. The phenotypic variability observed in DC, even among individuals with identical mutations like A353V, prompts consideration of modifying factors. Genotype-phenotype correlations across different populations remain complex, though certain mutations, including A353V in DKC1, are consistently linked to more severe disease manifestations. Beyond genetic background, environmental and treatment-related factors may influence disease expression. Chronic oxidative stress is implicated in accelerating telomere shortening and may exacerbate disease progression.^[21]^ Conversely, treatment interventions can modulate the phenotype. This underscores that the clinical course in DC is not solely genetically predetermined but may be shaped by a combination of exogenous and therapeutic influences.

**Table 1:** Vary clinical symptoms in patients with the A353 mutation in DKC1 previously reported.

No.	Gender	Age	Clinical symptoms					Inheritance	Ref.
			Reticular hyperpigmentation	Nail dystrophy	Oral leucoplakia	BMF	Other		
1	M	4	+	+	+	+	IUGR, dysphagia, epiphora	De novo	^[11]^
2	M	6	+	+	+	+	–	Maternally inherited*	^[12]^
3	M	NA	+	+	+	+	–	Maternally inherited*	^[12]^
4	M	10	+	+	+	−	Dysphagia, epiphora	Maternally inherited*	^[13]^
5	M	16	+	+	+	−	–	Maternally inherited*	^[13]^
6	NA	9	+	+	+	+	Epiphora, alopecia	NA	^[14]^
7	NA	7	+	+	+	+	Developmental delay	NA	^[14]^
8	M	8 mo	NA	NA	NA	+	Developmental delay, immunodeficiency, microcephaly, ataxia, watery diarrhea	NA	^[10]^
9	M	12	+	+	+	−	Epiphora	NA	^[15]^
10	M	24	+	+	+	+	Short stature, interstitial lung disease, dysphagia, hypogonadism, alopecia, hyperhidrosis	Maternally inherited*	^[16]^
11	M	NA	+	+	+	−	−	Maternally inherited*	^[16]^
12	M	36	+	+	+	+	Interstitial lung disease, pulmonary hypertension	De novo	^[17]^
13	M	60	+	+	+	−	–	Maternally inherited	^[17]^
14	M	2	+	+	+	+	IUGR, immunodeficiency, cerebellar hypoplasia	Maternally inherited	^[18]^
15	M	17	+	+	+	−	–	Maternally inherited*	^[19]^
16	M	22	+	+	+	−	–	NA	^[19]^
17	M	7	−	+	+	+	Dysphagia	De novo	^[20]^
18	M	10	+	+	+	+	Epiphora, dysuria	Maternally inherited	Index case

BMF = bone marrow failure, IUGR = intrauterine growth retardation, NA = not available.

* Confirmed de novo mutation in mother (maternal grandparents negative).

The classic mucocutaneous triad – reticulate skin hyperpigmentation, nail dystrophy, and oral leukoplakia – represents the primary clinical manifestation of DC.^[22]^ Reticulate pigmentation occurs in 90% of patients, and is often accompanied by skin atrophy and telangiectasia, predominantly affecting the face, neck, and chest. Hyperhidrosis and palmar/plantar hyperkeratosis are also frequently observed. Oral leukoplakia is present in about 80% of cases and may involve not only the oral mucosa but also the conjunctiva (causing epiphora), gastrointestinal tract (resulting in dysphagia), and urogenital tract (leading to dysuria). Notably, SCC develops in up to 30% of patients within leukoplakia-affected areas between the ages of 10 and 30, underscoring the need for regular surveillance and biopsy.^[23]^ Nail dystrophy, reported in 90% of cases, typically presents as ridging, longitudinal splitting, and eventual regression or shedding, sometimes accompanied by chronic paronychia.^[24]^ As a multisystem disorder, DC is also associated with BMF in 70 to 80% of patients, pulmonary complications such as fibrosis in 80%, and malignancies – including SCC and Hodgkin lymphoma – in approximately 10%.^[25]^ Other systemic complications may include hepatic dysfunction and gastrointestinal or genitourinary tract involvement. In its severe variant form, DC presents with HHS, characterized by intrauterine growth restriction, cerebellar hypoplasia, microcephaly, immunodeficiency, pancytopenia, and early mortality before overt mucocutaneous symptoms arise.^[11]^

The classic triad may not manifest simultaneously, and initial symptoms often involve extramucocutaneous systems, further complicating diagnosis. In the present case, the patient initially presented with thrombocytopenia and was misdiagnosed with aplastic anemia. It was only upon subsequent evaluation that skin and nail abnormalities were recognized, raising suspicion for DC – ultimately confirmed by genetic testing. This sequence highlights the essential role of a comprehensive physical examination in identifying this disorder. In addition to clinical assessment, telomere length measurement serves as an important diagnostic tool. Patients with DC typically exhibit significantly shortened telomeres, which can be assessed using methods such as PCR, FISH, and TRF.^[26,27]^ This approach is particularly useful when genetic testing is inconclusive. Although identification of a mutation in telomerase-related genes (e.g., DKC1, TERT, TERC, TINF2) confirms the diagnosis, a significant proportion of DC patients lack a detectable pathogenic variant. In such cases, markedly short telomeres provide strong supportive evidence for DC. Furthermore, telomere length assessment carries therapeutic implications, as DC patients exhibit heightened sensitivity to DNA-damaging agents used in chemotherapy and HSCT conditioning regimens. Although telomere length analysis was not performed in our case due to technical limitations, the presence of the characteristic clinical triad combined with identification of a pathogenic DKC1 mutation remains sufficient for a definitive diagnosis of DC.

Treatment options for DC remain limited. Immunosuppressants, glucocorticoids, and recombinant hematopoietic cytokines demonstrate suboptimal efficacy in managing DC-related complications.^[28]^ Specifically, BMF caused by DC typically shows poor response to these conventional agents. In this context, androgen therapy has emerged as a valuable medical intervention. The therapeutic mechanism is believed to involve upregulation of telomerase component expression, particularly TERT.^[29,30]^ Clinical evidence supports the efficacy of androgens in DC, with studies reporting hematologic responses in approximately 50 to 70% of patients, frequently achieving transfusion independence.^[31]^ In the present case, danazol was selected considering this favorable efficacy profile, the patient’s clinical stability, and as a strategy to defer or avoid the significant risks and costs of HSCT. However, potential adverse effects, particularly hepatotoxicity, necessitate close monitoring during treatment. HSCT remains the only curative treatment for DC, but it is associated with significant transplant-related toxicity.^[32]^ Reports indicated reduced survival in DC patients undergoing transplantation due to DNA damage induced by conditioning regimens involving radiation, busulfan, or high-dose cyclophosphamide.^[33]^ Reduced-intensity conditioning (RIC) protocols improved transplantation success rates and mitigated toxicity.^[33]^ Fludarabine, with its potent T-cell immunosuppressive properties, has become a preferred agent for RIC-HSCT. However, RIC-HSCT has been associated with neurological complications and increased tumor risk, necessitating strategies to minimize radiation-induced DNA damage. When considering HLA-matched sibling donors, including identical twins, it is essential to confirm the absence of DC manifestations and evaluate donors for genetic mutations and telomere length.

Oxidative stress plays a pivotal role in the pathogenesis of DC. Pharmacological interventions targeting oxidative stress have shown efficacy, with vitamin E demonstrating promising results in clinical trials.^[21]^ Furthermore, the sirtuin family of proteins (SIRT1–SIRT7) has emerged as a potential therapeutic target for DC. These proteins participate in various cellular processes including aging, inflammation, epigenetics, cancer, and cell cycle regulation. SIRT6, in particular, has been identified as a potential target for DC therapy due to its function as a deacetylase regulator at telomeric ends.^[34]^ Advances in genetic research also suggest gene therapy as a potential definitive treatment. The prognosis of DC varies considerably, ranging from infant mortality to survival into the seventh decade, with primary causes of death including BMF, malignancies, and pulmonary fibrosis.

## 5. Strengths and limitations

The case report’s strength lies in its demonstration of a systematic diagnostic pathway, integrating clinical suspicion with genetic confirmation, and its emphasis on multidisciplinary care involving hematology, genetics, and reproductive medicine. The successful use of prenatal diagnosis to prevent disease transmission further enhances its educational relevance. A notable limitation, however, was the inability to perform telomere length measurement – a supportive diagnostic assay for DC – due to technical constraints. Despite this, the diagnosis was unequivocally established using standard clinical and genetic criteria.

## 6. “Take-away” lessons

This case underscores the critical importance of considering rare genetic disorders, such as DC, in patients presenting with isolated cytopenias – particularly thrombocytopenia – especially when accompanied by atypical or evolving clinical signs. It reinforces the central role of genetic testing in confirming telomere biology disorders, as an early diagnosis allows for timely surveillance and management of multisystem complications. Moreover, this report exemplifies the clinical and ethical value of comprehensive genetic counseling. By identifying the pathogenic variant in the mother and facilitating prenatal testing, this process enabled informed reproductive decision-making and prevented the transmission of a severe, life-limiting condition.

## Author contributions

**Writing – original draft:** Ruifan Wen.

**Writing – review & editing:** Jidong Tian.
